# Streptococcus pneumoniae Coinfection in COVID-19: A Series of Three Cases

**DOI:** 10.1155/2020/8849068

**Published:** 2020-12-10

**Authors:** Chaitanya Pal, Paulina Przydzial, Ogechukwu Chika-Nwosuh, Shrey Shah, Pratik Patel, Nikhil Madan

**Affiliations:** ^1^Department of Medicine, Newark Beth Israel Medical Center, New Jersey 07112, USA; ^2^Division of Pulmonary and Critical Care Medicine, Department of Medicine, Newark Beth Israel Medical Center, New Jersey 07112, USA

## Abstract

Bacterial coinfections are not uncommon with respiratory viral pathogens. These coinfections can add to significant mortality and morbidity. We are currently dealing with the SARS-CoV-2 pandemic, which has affected over 15 million people globally with over half a million deaths. Previous respiratory viral pandemics have taught us that bacterial coinfections can lead to higher mortality and morbidity. However, there is limited literature on the current SARS-CoV-2 pandemic and associated coinfections, which reported infection rates varying between 1% and 8% based on various cross-sectional studies. In one meta-analysis of coinfections in COVID-19, rates of Streptococcus pneumoniae coinfections have been negligible when compared to previous influenza pandemics. Current literature does not favor the use of empiric, broad-spectrum antibiotics in confirmed SARS-CoV-2 infections. We present three cases of confirmed SARS-CoV-2 infections complicated by Streptococcus pneumoniae coinfection. These cases demonstrate the importance of concomitant testing for common pathogens despite the need for antimicrobial stewardship.

## 1. Introduction

Severe acute respiratory syndrome coronavirus 2 (SARS-CoV-2) is a novel, zoonotic, positive-sense, single-stranded RNA beta coronavirus which is responsible for the current global pandemic of coronavirus 19 (COVID-19) pneumonia. With depleting resources and continued spread of the disease, it is easy to forget screening for most common causes of pneumonia, especially when they present as seemingly less important coinfections. We describe three cases of SARS-CoV-2 infection complicated by Streptococcus pneumoniae coinfection, their presentation, radiographic changes, and clinical outcomes.

## 2. Case 1

**Figure 1:**
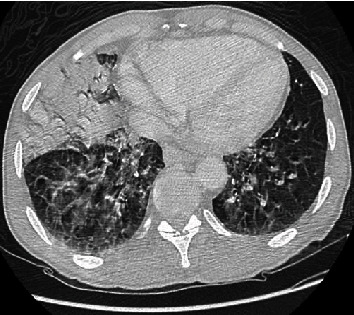
CT chest showing bilateral ground-glass opacities and right middle lobe consolidation.

A 51-year-old male with a past medical history of emphysema, opioid abuse, and HIV not on antiretroviral therapy presented to the ED via EMS due to alcohol intoxication and right-sided pleuritic chest pain. The patient was intoxicated upon presentation but was not able to ascertain any history of fevers, cough, or shortness of breath. His temperature was 98.8 degrees Fahrenheit, blood pressure 143/82 mmHg, heart rate 84 bpm, respiratory rate 21, and oxygen saturation 82% breathing room air. He appeared comfortable at rest on oxygen. The respiratory exam revealed bilateral rhonchi with poor bilateral air entry. A CT chest revealed bilateral ground-glass opacities as well as a right middle lobe consolidation (Figure 1). His oxygenation status progressively worsened requiring a nonrebreather mask at 15 liters per minute. The patient was empirically treated for community-acquired pneumonia with piperacillin and tazobactam. Complete blood count on admission showed white blood cells of 5.1 with a normal differential. The comprehensive metabolic panel was normal except for an elevated AST and ALT at 108 and 78, respectively. Arterial blood gas showed a pH of 7.31, pCo_2_ of 47 mmHg, and pO_2_ of 66 mmHg. Procalcitonin was elevated at 0.13 ng/mL. On the second day of admission, he tested positive for SARS-CoV-2 via nasal PCR as well as Streptococcus pneumoniae on lower respiratory culture. The patient's antibiotic regimen was changed to doxycycline. Despite a low CD4 count of 10.2, the patient had no other opportunistic infections. He did not receive hydroxychloroquine due to concurrent use of methadone and concerns for a prolonged QT interval. He had fever of 100.8 and 100.7 degrees Fahrenheit on day 3 and day 5 of admission, respectively, but remained afebrile during his hospital stay of 8 days. Oxygen therapy with a nonrebreather mask was deescalated to 2 L nasal cannula progressively through his hospital stay. He was discharged home in stable condition without any oxygen requirements.

## 3. Case 2

**Figure 2:**
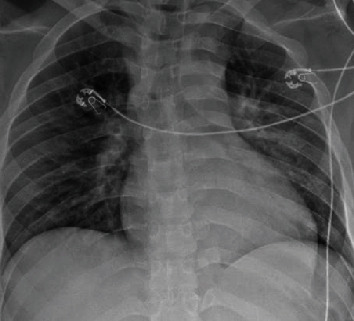
Chest X-ray showing bilateral infiltrates.

A 62-year-old male with a past medical history of hypertension, diabetes mellitus, and coronary artery disease presented to the ED with complaints of generalized weakness, myalgia, exertional dyspnea, pleuritic chest pain, and dry cough of 5-day duration. On admission, he had a temperature of 102.8 degrees Fahrenheit, blood pressure of 150/79, heart rate of 120 bpm, and respiratory rate of 20 with oxygen saturation of 90% on room air. He was comfortable at rest with a normal physical exam. He was treated with supplemental oxygen via a 4 L nasal cannula. Complete blood count on admission showed white blood cells of 11,200/mcL with an elevated neutrophil count. He was noted to be in acute renal failure with a BUN and creatinine of 28 mg/dL and 1.69 mg/dL, respectively. Procalcitonin was elevated at 2.18 ng/mL. Chest X-ray showed bilateral lower lobe infiltrates (Figure 2), and he was empirically treated for community-acquired pneumonia with doxycycline. On the second day of admission, he tested positive for SARS-CoV-2 via PCR of nasal swab as well as Streptococcus pneumoniae urine antigen. He was started on Plaquenil and continued antibiotic therapy. On the second day of admission, he no longer required supplemental oxygen, and he was successfully discharged home with 5 days of oral doxycycline.

## 4. Case 3

**Figure 3:**
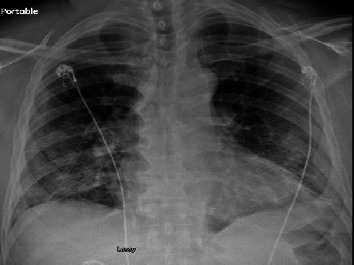
Chest X-ray showing diffuse bilateral infiltrates.

A 21-year-old male with a past medical history of type 1 diabetes mellitus presented to the ED with weakness, fevers, cough, and pleuritic chest pain as well as elevated blood glucose readings noted at home. On admission, his temperature was 100.2 degrees Fahrenheit, blood pressure of 135/97 mm Hg, heart rate of 131 bpm, respiratory rate of 30 bpm, and oxygen saturation of 95% breathing room air. Complete blood count on admission showed elevated white blood cells of 27,900/mcL. The basic metabolic panel showed a blood glucose of 408 mg/dL, an elevated anion gap, and serum bicarbonate of 10 mmol/L. He was diagnosed with diabetic ketoacidosis and treated with insulin drip and intravenous fluids. Chest X-ray (Figure 3) showed bilateral perihilar infiltrates; antibiotic therapy with ceftriaxone was started. For concerns of SARS-CoV-2 pneumonia, he was started on hydroxychloroquine. He improved within 24 hours and was discharged home 2 days later. After discharge, diagnostics revealed SARS-CoV-2 positivity and blood cultures growing Streptococcus pneumoniae. Our patient was called back to return to the hospital for the treatment of bacteremia and COVID-19 pneumonia. Ceftriaxone was resumed, and vancomycin was added while awaiting sensitivities. Hydroxychloroquine was continued to complete a 5-day course. He remained afebrile and hemodynamically stable throughout the hospital course of 4 days. He did not require supplemental oxygen therapy. After confirming, repeated blood cultures were negative; he was discharged home on oral penicillin treatment.

## 5. Discussion

Bacterial coinfections are a major cause of morbidity and mortality in respiratory viral infections. Lung tissue samples from the 1918 influenza pandemic suggest that the majority of the estimated 20–60 million deaths were attributable to concomitant bacterial infections, rather than from direct effects of the virus [1]. Although the incidence rate of bacterial coinfection in COVID-19 is currently unclear, the limited studies appear to suggest that coinfection rates are much lower than in previous pandemics [2]. Due to resource constraints, overburdened healthcare systems, and current evidence suggesting lower rates of coinfections, it is plausible that patients infected with COVID-19 are simply not being evaluated for coinfections. Thus, the true coinfection rate may be higher than what is currently suggested by the available literature. In a literature, albeit a very small study, it was noted that COVID-19-associated co and secondary infection had a prevalence of about 0.6% to 45% in the 10 studies conditioned [3]. In these articles, the organisms causing coinfection were as follows: M. pneumoniae, Legionella pneumophila, Streptococcus pneumoniae, and C. pneumoniae [3]. Another important factor is that there is currently no consensus on the direct effects of coinfection in COVID-19 on morbidity and mortality. Some studies have suggested more severe and complicated disease courses in cases of coinfection [2].

Streptococcus pneumoniae is a lancet-shaped, gram-positive, facultative anaerobic organism that has historically been the most common culprit pathogen causing community-acquired pneumonia worldwide. Although pneumococcal pneumonia can present in all populations from childhood to adulthood, it is more common in patients older than 65 years of age; those who smoke, abuse alcohol, and have asthma or COPD; or those who are asplenic [4]. This same demographic of patients has been studied in SARS-CoV-2 infection and has been associated with the worst prognosis. Pneumococcal pneumonia classically presents as a unilateral consolidation on chest radiographs but may also assume a bilateral, interstitial pattern. This bilateral pattern appears radiographically similar to pneumonia due to SARS-CoV-2 [5]. This poses a diagnostic challenge for clinicians amidst the current pandemic. Streptococcus pneumoniae C-polysaccharide antigen in urine has a high sensitivity and specificity in diagnosing pneumococcal pneumonia and can be used for quick and effective screening in all patients.

Among the patients presented in our case series, albeit a small population, shared features include early testing, diagnosis, and treatment for COVID-19 and pneumococcal coinfection, as well as successful discharge and patient recovery. All three patients tested positive for SARS-CoV-2 infection by nasal swab PCR testing, their streptococcal immunization was unknown, and no other organisms were recovered. Streptococcal infection was detected through either polysaccharide antigen testing, blood cultures, or sputum cultures. All the three patients had respiratory failure requiring oxygen supplementation. Radiological features varied throughout the three patients, one showing CT scan findings of discrete consolidation, and the other two with diffuse bilateral infiltrates on chest X-ray. Therapy for SARS-CoV-2 infection was limited to supportive therapy in one of the three patients, while the others received hydroxychloroquine. All three patients were successfully discharged home without any oxygen supplementation requirements after recovery. We propose that, despite the current literature showing lower rates, bacterial coinfection in the current pandemic when compared to previous viral pandemics, there is still a significant role in evaluating and empirically covering for coinfections. There may also be a role in emphasizing compliance of pneumococcal vaccinations in high-risk patients to avoid coinfection and improve morbidity and mortality.
